# An assessment of validity and responsiveness of generic measures of health-related quality of life in hearing impairment

**DOI:** 10.1007/s11136-013-0417-6

**Published:** 2013-05-26

**Authors:** Yaling Yang, Louise Longworth, John Brazier

**Affiliations:** 1 Health Economics Research Group, Brunel University, Uxbridge, UK; 2 Health Economics and Decision Science, School of Health and Related Research, University of Sheffield, Sheffield, UK

**Keywords:** Validity, Responsiveness, EQ-5D, HUI3, SF-6D, Hearing impairments

## Abstract

**Purpose:**

This review examines psychometric performance of three widely used generic preference-based measures, that is, EuroQol 5 dimensions (EQ-5D), Health Utility Index 3 (HUI3) and Short-form 6 dimensions (SF-6D) in patients with hearing impairments.

**Methods:**

A systematic search was undertaken to identify studies of patients with hearing impairments where health state utility values were measured and reported. Data were extracted and analysed to assess the reliability, validity (known group differences and convergent validity) and responsiveness of the measures across hearing impairments.

**Results:**

Fourteen studies (18 papers) were included in the review. HUI3 was the most commonly used utility measures in hearing impairment. In all six studies, the HUI3 detected difference between groups defined by the severity of impairment, and four out of five studies detected statistically significant changes as a result of intervention. The only study available suggested that EQ-5D only had weak ability to discriminate difference between severity groups, and in four out of five studies, EQ-5D failed to detected changes. Only one study involved the SF-6D; thus, the information is too limited to conclude on its performance. Also evidence for the reliability of these measures was not found.

**Conclusion:**

Overall, the validity and responsiveness of the HUI3 in hearing impairment was good. The responsiveness of EQ-5D was relatively poor and weak validity was suggested by limited evidence. The evidence on SF-6D was too limited to make any judgment. More head-to-head comparisons of these and other preference measures of health are required.

## Introduction

Economic evaluations using a cost-utility framework have been increasingly used to support decision-making in the allocation of health resources and priority setting [1–3]. Cost-utility analyses assess health care interventions in terms of the incremental cost per quality-adjusted life year (QALY) gained. QALYs combine length of life with a quality of life where the quality of life component is usually based on health state utility values [4].

One common way to obtain health state utility values is to use one of the generic preference-based measures (GPBMs) of health-related quality of life. Examples of the most commonly used GPBMs include the EuroQol 5 dimension (EQ-5D) [5, 6], Short-form 6 dimension (SF-6D) [7] and the Health Utilities Index (HUI3) [8]. For the purpose of economic evaluation, these measures have the advantage of reflecting the value people place on different dimensions or levels of health and enable comparisons of health outcomes to be made across conditions. EQ-5D has 5 health dimensions (mobility, self-care, usual activities, pain/discomfort and depression/anxiety). Each dimension has 3 levels of severity in the original version, and a version with 5 levels of severity has recently been developed [5, 9]. Derived from the Short-form 36 and Short-form 12 health questionnaires, the SF-6D has 6 dimensions (physical functioning, role limitation, social functioning, bodily pain, mental health and vitality), and each dimension has 4–6 severity levels. The HUI3 has 8 dimensions (vision, hearing, speech, ambulation, dexterity, emotion, cognition and pain), and each dimension has 5 or 6 severity levels. These measures differ in terms of the description of health. The HUI3 can be seen as a ‘within the skin’ measure of health and includes sensory dimensions such as vision, speech and hearing. EQ-5D and SF-6D focus more on how health impacts on functioning in life, but nonetheless there are important differences in coverage, such as EQ-5D not having vitality and SF-6D containing role and social functioning dimensions compared to usual activities in EQ-5D. Apart from the different descriptive systems, the measures also differ in terms of the methods used to estimate health state values.

Empirical evidence has confirmed that health state utility values obtained from these three GPBMs are different from each other [10–14]. In order to allow comparability between conditions, in the United Kingdom, the National Institute for Health and Clinical Excellence has prefers the use of a single GPBM, the EQ-5D [15]. However, GPBMs have attracted criticism for failing to capture important aspects of health and insensitive to the change of health states because one or more important dimensions of health relevant to a medical condition have been excluded [16–18] [19–21]. National Institute for Health and Clinical Excellence recognized that there may be specific circumstances in which the EQ-5D is not appropriate and offers some advice for these circumstances; however, it does not identify those areas where EQ-5D is inappropriate nor provide criteria to determine this.

More evidence on the performance of EQ-5D and other GPBMs is required for a wider range of conditions and/or treatments to demonstrate whether these measures are appropriate for these conditions in order to judge when alternative measures should be considered. The assessment of the validity and responsiveness of GPBMs is fraught with conceptual and empirical problems owing to the lack of a gold standard measure. However, by taking into account of a range of evidence on specific conditions in a systematic and transparent way, it is possible to judge the performance of the instruments [22]. This is important in order to help inform which measures should be included for the assessment of benefits of specific health interventions, or for interpreting the evidence from population studies that include such instruments. For example, recently in the United States, the National Health Measurement Study (http://www.healthmeasurement.org/NHMS.html) attempted to build a versatile ‘toolbox’ for this purpose, and EQ-5D, Short-form 36 version 2 (from which SF-6D can be derived) and HUI were included in the toolbox (http://www.healthmeasurement.org/NHMS.html).

Hearing impairment is one of the most common chronic health problems in Western society, in part due to the growth of the elderly population, affecting 15 % of the adult population [23]. Hearing loss affects a person’s ability to communicate, social participation, independence, employment and overall quality of life [23]. Previous research has suggested that health state utility values obtained from people with hearing impairments are different using different generic instruments [24]. A review of the evidence on the validity of GPBMs in hearing impairment has not been previously undertaken. The aim of this study was to systematically review the published literature to assess the reliability, validity and responsiveness of three key generic measures of health-related quality of life (EQ-5D, HUI3 and SF-6D) in people with hearing impairment.

## Methods

### Search strategy and data identification

The objective of the literature review was to identify published papers reporting evidence of the performance of EQ-5D, HUI3 and SF-6D in patients with hearing impairments.

A broad search was conducted to identify studies reporting EQ-5D, SF-6D and HUI3 to examine the health-related quality of life of patients with a hearing impairment. BIOSIS, CINAHL, EMBASE, MEDLINE, PsychINFO and Web of Science electronic databases were searched. The database available from the EuroQol Group Website was also searched but comparable databases for HUI3 and SF-6D are not available. The search focused on key words search, including ‘hearing impairment/disorder’, ‘euroqol/EQ-5D’, ‘hui3’ and ‘sf6d’, all with alternative spellings. The search strategy is presented in Appendix 1. The criteria for inclusion were that the study population had a hearing impairment, the study reported at least one from the EQ-5D, SF-6D or HUI3 and reported another measure of quality of life (generic- or condition-specific) or a measure of clinical severity, or direct valuation of health. Papers only reporting EQ-VAS (EuroQol Visual Analogue Scale) scores were excluded as the main interest was the descriptive systems and utility indices of the three measures. Papers that only used vignettes or own health state valuations, and not one of the three generic measures, were excluded. There was no restriction relating to the type of study. Due to resource limitations, only English language studies were reviewed.

### Analytic strategy

#### Data extraction

Considering the aim of the study and reviewing forms used for similar studies in other disease areas [17, 25], a template was developed to extract data in a standardised format including the following:

- Study characteristics—country, type of hearing impairment, disease or treatment stage, any treatment given, study design;
- Participant characteristics—number of participants, age, gender, ethnicity, missing data;
- Instruments used—EQ-5D/SF-6D/HUI3, other generic measures of health-rated quality of life, condition-specific health-related quality of life measures and clinical measures of disease severity, patient’s own health state valuations (e.g. Visual Analogue Scale (VAS), Time trade-off (TTO) and Standard Gamble (SG));
- Health state utility values—mean of utility index, scoring algorithm;
- Construct and convergent validity—methods of assessment and results;
- Responsiveness—methods of assessment and results.
- Reliability—methods of assessment and results.

#### Quality assessment of studies

For the review, of the most importance was the relevance of the study in terms of the patient population and inclusion of evidence to answer our research question. Nevertheless, the quality of studies was assessed by examining study design, recruitment process, sample size and the extent of missing data reported. The intention of the assessment of quality was not to exclude relevant studies. Rather, it gives some indications of quality to assist with the interpretation of the findings. It should be noted that some studies may be of high quality for their research question, but provide limited information for the assessment of validity, reliability and responsiveness. For example, a case–control study may be well designed and conducted, but offer information limited to the presence or absence of hearing problems, and not detailed information on the severity of the condition.

#### Assessment of validity

Validity is defined as how well an instrument measures what it was intended to measure [22, 26]. Ideally, validity would be assessed by comparing an instrument to an established gold standard. However, in the case of health-related quality of life, no gold standard measure exists. Therefore, construct validity was assessed by making comparisons with other measures of quality of life and disease severity, and assessing the totality of that evidence to see whether the results from the GPBMs reflect the patterns in scores seen in those other measures [22].

A common test to identify construct validity is the ‘known group’ method [22]. This is determined by the degree to which an instrument can demonstrate different scores for groups know to vary on the variables being measured. In this study, health state utility values are compared between groups of patients that are defined in terms of disease severity and trends in the pattern of utility, statistical tests (e.g. *t* test) and regression were used for assessment. The patient population could be stratified on the basis of a clinical indicator or a health-related quality of life measure (generic- or condition-specific). A less stringent test of construct validity is to define groups using a case–control analysis where scores of patient group and non-patient groups or general population are compared.

Another type of construct validity is known as convergent validity [22]. This is defined as the extent to which one measure correlates with another measure of the same concept (although this measure is not regarded as gold standard). In this review, the extent to which EQ-5D, SF-6D or the HUI3 correlated with other measures of hearing problems or health-related quality of life was examined based on statistics including correlation coefficients or regression analysis with hearing-specific health-related quality of life measures or measures of hearing loss.

#### Assessment of responsiveness

Responsiveness is the ability to measure change. A pre/post-intervention study which reports EQ-5D, SF-6D or the HUI3 and another valid measure of health change would allow the responsiveness of a measure due to change in health status to be identified. As with the tests of validity, it is important to consider whether the measures of health change that used for comparison are valid themselves. In addition, it is important to consider whether other health changes not directly related to the condition could have impacted upon health-related utility (for example, side effects of treatment).

#### Assessment of reliability

The reliability of a measure is defined as its ability to reproduce results when measurements are repeated on an unchanged population [22]. Reliability can be measured by retesting and reporting either the correlation or difference between estimates. For this study, the measures were considered reliable if they demonstrated no change in health-related quality of life when the other reference measures also demonstrated no change in health.

#### Presentation of data

Data were presented in a series of summary tables as well as brief text, providing information on characteristics and quality assessment of included studies, the measures included, methods and result for validity assessment, methods and results for responsiveness assessment. At the end, a table providing an overview of performance of EQ-5D, HUI3 and SF-6D was presented recording the findings as a ‘√’ if the evidence supported the statement, or ‘x’ if the evidence did not support the statement, or ‘?’ if the evidences were mixed and conclusion could not be made, or ‘N/R’ if no information was reported. If the pattern and direction of EQ-5D were consistent with other measures in terms of difference between groups or change over time, this was considered as supporting evidence. Correlation coefficients were grouped as small (<0.3), moderate (0.03–0.5) and strong (>0.5), and a significant predictor of regression was recorded as ‘√’.

## Results

### Search results

**Fig. 1:**
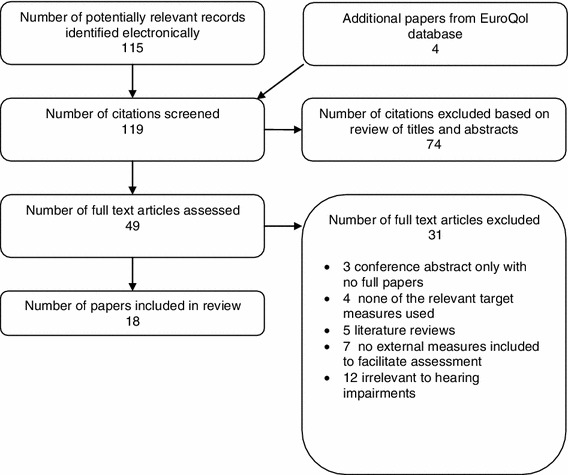
Flow diagram showing selection of studies

Bibliographic searching was completed in July 2010. The search strategy identified 119 articles. After reviewing titles and abstracts, 70 papers were excluded. Forty-nine papers were reviewed in full, and a further 31 were excluded and 18 papers were included in the final review (see Fig. 1). Papers were included if they provided sufficient evidence to assess the validity, responsiveness and reliability of EQ-5D, HUI3 and SF-6D. However, the paper did not have to have been designed for this purpose. Papers were excluded if they did not include one of the generic measures of interest. Papers were also excluded if validity or responsiveness could not be assessed because no other clinical or quality of life measures were included, and differences over time or between interventions were not reported. Since the focus of the review is preference-based measures, papers only reporting Visual Analogue Scale scores were also excluded.

### Quality assessment and characteristics and of included studies

Most studies were not designed to specifically assess the validity, reliability and responsiveness of the instruments, but all provided data in sufficient detail to allow an assessment to be made. A range of recruitment procedures was noted in the studies included in the review. Some were cross-sectional observational studies [27, 28] but the majority were prospective or retrospective before–after studies [29–32]. Studies had well-defined inclusion/exclusion criteria in recruitment. Sample size ranged from 20 to 3,272 with most of studies had around 100 participants but two studies only had around 20 participants [29, 33]. For longitudinal studies, no study had extremely high levels of missing data. The reporting in these papers was reasonably clear. After quality assessment, no studies were excluded from the review.

The main characteristics of the 18 papers included in this review are shown in Table 1. The four papers by Joore et al. [31, 32]; Joore [34, 35] reported the results of one specific study and similarly the two papers by Vuorialho et al. [36] reported a single study, resulting in 14 studies in total. The studies were undertaken in a range of countries, including United Kingdom, the Netherlands, USA, Canada and Finland. Some studies recruited patients with specific hearing problems, for example, Large Vestibular Aqueduct Syndrome [29], profoundly deaf or conductive or mixed hearing loss [37, 38]. Twelve papers reported hearing loss of their sample using clinical indicators such as the better ear unaided pure-tone average. As shown in Table 1, the level of hearing loss varied between studies.

**Table 1:** Characteristics of the 13 studies included in the review

Author, Year	Country	Hearing disorder	Intervention	Study design	Number of participants	Mean age (SD or range)	Female (%)
Barton et al. [24]	United Kingdom	Hearing impaired	Hearing aid (analogue vs. digital signal processing)	Prospective before–after study	609	68.4	43 %
Barton et al. [41]	United Kingdom	Hearing impaired	Cochlear implant	Cross-sectional	3,272	6 (at CI implantation)	N/R
Damen et al. [43]	The Netherlands	Post-lingual deaf adults.	Cochlear implant	Prospective before and after.	37 (G1) 17 (G2) 29 (G3)	55.1 (SD 16, G1), 50.5 (SD 21.9, G2), 61.5 (SD 13.1, G3)	54 % (G1), 50 % (G2), 32 % (G3)
Gruters et al. [30]	The Netherlands	Hearing impaired	Hearing aid		337	69.6 (SD 8.9)	40 %
Hol et al. [38]	The Netherlands	Conductive or mixed hearing loss	Bone-anchored hearing aid	Prospective before–after study	56	52.9 (total, 24–82), 47.9 (ACHA, 24–73), 62 (CBHA, 42–82)	61 % (total), 67 % (ACHA), 55 % (CBHA)
Joore et al. [31, 32]; Joore [34, 35]	The Netherlands	First-time hearing aid users	Hearing aid	Prospective before–after study	126	69 (29–96)	50 %
Palmer et al. [42]	Canada and United States	Severely to profoundly hearing-impaired adults	Cochlear implant	Prospective before–after study	62	56 (CI, SD 15.4), 49 (non-CI, SD 14.5)	54 % (CI) 84 % (non-CI)
Vuorialho et al. [36, 39]	Finland	First-time hearing aid user over 60	Hearing aid	Prospective before–after study	101	77 (Median, 61–87)	50 %
Lee et al. [33]	South Korea	Post-lingual deaf adults	Cochlear implant	Retrospective before–after study	26	49.6 (SD 10.9)	36.4 %
Bichey et al. [29]	USA	Large vestibular aqueduct syndrome	Cochlear implant vs. hearing aid	Retrospective before–after study	20	44.3 (Median, CI, 9.9–75.6); 22.5 (Median, HA, 8.6–65.1)	N/R
Cheng et al. [37]	USA	Profoundly deaf	Cochlear implant	Retrospective study	78 (VAS group), 40 (TTO group), 22(HUI3 group)* 22(HUI3 group)*	7.5 (VAS), 7.4 (TTO), 10 (HUI3) 38.3 (parents)	46 % (child), 89 % (parent)
Sach and Barton [40]	United Kingdom	Hearing-impaired children and their parents	Unilateral cochlear implant	Retrospective before–after study	222*	9.26 (SD 3.63)	49.1 %
Lovett et al. [27]	United Kingdom	Profoundly deaf.	Cochlear implant (bilateral and unilateral)	Cross-sectional observational study	50	7.2	40 % (unilateral) 53 % (bilateral)
Smith-Olinde et al. [28]	USA	Permanent childhood hearing loss	Cochlear implant	Cross-sectional study	146	7.3 (SD 1.9)	48.5 %

*SD* standard deviation, *ACHA* used air-conduction hearing aid, *CBHA* conventional bone-conduction hearing aid, *VAS* Visual Analogue Scale, *TTO* Time trade-off, *HUI3* Health Utility Index 3, *N/R* not report

* Involve deaf children and their parents

Five studies included young children with hearing impairments (mean ages of the samples ranged from 7.3 to 9.3 years old), and the remaining studies included adults in their studies with most focussing on older adults over 60 years. The studies involving children used parents or caregivers as proxies to assess health-related quality of life of children.

### Measures and clinical indicators used in the studies included

Table 2 summarises the measures which have been used in the 18 papers. For the three generic preference-based measures of interest, 11 papers reported EQ-5D, 11 reported HUI3 and 1 used the SF-6D (alongside EQ-5D and HUI3). Among those studies that used EQ-5D, most reported the EQ-5D index based on the tariff of UK population values. In two cases, it was unclear which tariff of population values had been used. Three papers also reported responses on the five EQ-5D dimensions alongside the utility indices using tariff [31, 34, 39]. One study [30] also compared EQ-5D results using the UK and Dutch tariff. Only the Canadian tariff was used to value the HUI3. Two studies used Quality of Well-being (QWB, another generic preference-based measure of health) alongside EQ-5D or HUI3.

**Table 2:** Measures reported in the papers

Author, Year	Generic utility measures			Direct valuations	Rating scales	Hearing-specific measures	Clinical indicators
	EQ-5D	HUI3	SF-6D	TTO	VAS	–	–
Barton et al. [24]	√	√	√	–	–	–	–
Barton et al. [41]		√					AHL
Gruters et al. [30]	√	√	–	–	–	–	BEPTA
Lee et al. [33]	√	√	–	√	√	–	–
Bichey et al. [29]	–	√	–	–	–	–	PTA
Cheng et al. [37]*	–	√		√	√	–	–
Damen et al. [43]	–	√	–	–	–	NCIQ	NVA and AN test
Lovett et al. [27]	–	√	–	–	√	SSQ	–
Palmer et al. [42]	–	√	–	–	–	–	NU-6; Audiologic mean score for CID sentence recognition.
Smith-Olinde et al. [28]	–	√	–	–	–	–	BEPTA
Hol et al. [38]	√	–	–	–	EQ-VAS	HHDI	–
Joore et al. [31]	Index and responses	–	–	–	VAS and EQ-VAS	ADPI	–
Joore et al. [32]	Index and responses	–	–	–	VAS and EQ-VAS	–	–
Vuorialho et al. [36]	Index and responses	–	–	–	√	HHIE-S	BEHL, SRT, WRS
Joore et al. [34]	√	–	–	–	√ and EQ-VAS	–	–
Joore et al. [35]	√	–	–	–	√ and EQ-VAS	HHIE-S and hearing aid satisfaction/use	–
Sach and Barton [40]	√	–	–	–	EQ-VAS and quality of life VAS	–	–
Vuorialho et al. [39]	√	–	–	–	EQ-VAS	HHIE-S, hearing aid satisfaction	–

*EQ*-*5D* EuroQol 5 dimensions, *HUI3* Health Utility Index 3, *SF*-*6D* Short-form 6 dimensions, *TTO* Time trade-off, *VAS* Visual Analogue Scale, *AHL* average of pure-tone air-conduction thresholds at the frequencies 0.5, 1, 2 and 4 kHz in the better hearing ear, *BEPTA* (better ear pure-tone average hearing loss for the frequencies 1,000, 2,000 and 4,000 Hz, *PTA* ear-specific and bilateral four-frequency pure-tone averages, *NCIQ* the Nijmegen cochlear implant questionnaire, *NVA* test (an open speech recognition test), *AN* test (assess suprasegmental identification, a closed-set spondee identification test and a closed-set number of syllables test), *SSQ* speech, spatial and qualities of hearing scale for parents, *NU-6* Northwestern University 6 word test, *CID* central institute for the deaf, *EQ*-*VAS* euroqol Visual Analogue Scale, *HHDI* Hearing Handicap and Disability Index, *ADPI* Audiological Disabilities Preference Index, *HHIS*-*S* Hearing Handicap Inventory for the elderly, *BEHL* better ear hearing levels over the frequencies 0.5–4 kHz, *SRT* speech reception thresholds, *WRS* word reception scores (%), *APHAB* abbreviated profile of hearing aid benefit

* Parents were proxies

A total of 11 papers also reported Visual Analogue Scale (VAS) results including: EQ-VAS, a general health VAS, a hearing-specific VAS and a general quality of life VAS. In total, 7 papers reported the EQ-VAS and used imaginable best and worst imaginable health as anchors. Among them, 4 publications related to a single study reported the results of a hearing-specific VAS [31, 32, 34, 35] using ‘deaf’ and ‘perfect sense of hearing’ as the anchors. One study [40] regarded hearing impairment as having an effect beyond health or HRQoL, so alongside EQ-VAS, another VAS using the best/worst quality of life as anchors were also reported. The remaining four papers reported a general health VAS and among them, one used ‘death’ and ‘the imaginable best health’ as anchors, one used ‘imaginable worst/best health’ as anchors and for the other two, anchors were not clearly reported.

Time trade-off (TTO) values were obtained without the use of generic measures in 2 studies [33, 37]. The study by Cheng et al. used parents as proxies to assess their deaf children’s utility and the TTO compared two alternatives: one being in the current health state without hearing aid for remaining life expectancy and another alternative being in perfect health for a shorter time period. The values in the study by Lee et al. were not actual TTO values but predictions from VAS transformed using a power formula.

A total of 9 studies employed self-reported hearing-specific health-related quality of life measures. This included 3 studies using the Hearing Handicap Inventory for the Elderly, 2 using the Hearing Handicap and Disability Index, and 1 using the Nijmegen cochlear implant questionnaire, the Speech Spatial and Qualities of hearing scale for parents, Amsterdam Inventory and Audiological Disability Preference Index which is a hearing-specific preference-based measure derived from Amsterdam Inventory. Six studies reported clinical indicators to indicate severity of hearing impairment, including pure-tone average for the best or worst ear without hearing aid and speech identification tests.

### Reliability of GPBMs in hearing impairment

The review found little evidence on the reliability assessments of EQ-5D, HUI3 and SF-6D in hearing impairment. No papers reported conducting test–retest experiments. Although not specifically for test–retest reliability purposes, one study [34] reported EQ-5D responses and VAS indices at baseline and asked respondents to recall them 3 months after hearing aid fitting. They did not find any significant difference between the baseline assessment and the recalled assessment of baseline health for EQ-5D.

### Construct validity of GPBMs in hearing impairment

Out of the 18 papers include in the review, 7 papers provided information to enable an assessment of the EQ-5D, HUI3 or SF-6D, although most studies were not designed to examine the validity of these measures. The results are summarised in Table 3.

**Table 3:** Summary of validity of EQ–5D, HUI3 in hearing impairments

Study	Instrument	Assessment	Methods	Summary of results
Barton et al. [24]	HUI3/EQ-5D/SF-6D	Convergence	Correlations between measures	Moderate to strong correlations were found between HUI3, EQ-5D and SF-6D.
Barton et al. [41]	HUI3	Known groups(severity) Convergence	HUI3 scores and severity groups defined by AHL level	HUI3 mean scores were different between moderate, severe, profound1, profound2 and implanted groups (significance not reported) CI (grouped by age at implantation and duration of use), AHL, gender were significant predictor of HUI3 (*p* < 0.01)
Bichey et al. [29]	HUI3	Known groups (severity)	HUI3 scores and PTA (presented by CI and HA group	HUI3 mean scores: 0.82 (CI) versus. 0.62 (HA) Consistent with PTA. No statistical test reported.
Damen et al. [43]	HUI3	Convergence	Spearman rho correlations between mean score of different measures at the follow-up	Correlation coefficients: 0.33 (HUI3 and AN test, *p* < 0.05) 0.39 (HUI3 and NVA test, *p* < 0.05) 0.48 (NCIQ and AN test, *p* < 0.05) 0.32 (NCIQ and NVA test, *p* < 0.05)
Lovett et al. [27]	HUI3	Known groups (severity)	HUI3 index scores and SSQ, VAS scores presented by unilateral and bilateral implantation groups	A significant difference (*p* < 0.05) in favour of bilateral (SSQ); No significant (*p* = 0.2) differences detected (HUI3 and VAS)
Palmer et al. [42]	HUI3	Known groups (severity)	HUI3 index scores presented by CI and non-CI implant groups at enrolment, 6 months and 12 months after CI implant.	Difference between CI and non-CI groups by HUI3: Not significant (baseline) and significant (*p* < 0.1) difference (0.76 for CI and 0.58 for non-CI) at both 6 and 12 months after intervention.
Smith-Olinde et al. [28]	HUI3	Known groups (severity)	HUI3 utility index presented by 4 groups defined by the degree of hearing loss	Both HUI3 and QWB scores declined with the degree of hearing loss where a greater extent for HUI3 than QWB. No statistical significance was presented
Gruters et al. [30]	EQ-5D (UK and Dutch tariff), HUI3	Known groups (age gender and severity) Convergence	Utility scores compared between age, gender (EQ-5D) and clinically distinctive groups (HUI3) Agreements between utility scores by Kendall’s Tau correlation and ICC	Significant differences detected: Age and gender (by EQ-5D); Clinical groups (by HUI3). Kendall’s Tau correlations: 0.36–0.41 (between EQ-5D with UK or Dutch tariff and HUI2, HUI3) ICC: 0.44–0.51 (between utility measures)
Sach and Barton [40]	EQ-5D	Known groups (through regressions)	Multiple linear regression was estimated between the child’s EQ-5D scores and CAP, as well as other variables)	Statistically significant coefficients (*p* < 0.05) for children with/without additional disabilities, gender, more severe deaf condition (measured by CAP); Non-statistical significant coefficients (*p* > 0.05) for children having mild deaf (in the top three levels of the CAP) and other socio-economic factors.

*HUI3* Health Utility Index 3, *AHL* average of pure-tone air-conduction thresholds at the frequencies 0.5, 1, 2 and 4 kHz in the better hearing ear, *PTA* pure-tone average, *CI* cochlear implant, *HA* hearing aid, *NCIQ* the Nijmegen cochlear implant questionnaire, *SSQ* speech, spatial and qualities of hearing scale for parents, *VAS* Visual Analogue Scale, *QWB* Quality of well-being scale, *EQ*-*5D* euroqol 5 dimensions, *ICC* intraclass correlation, *CAP* categories of auditory perception

#### ‘Known group’ differences

Seven studies presented data to allow an assessment of ‘known group’ differences where the groups were defined by severity of hearing loss. Using ANOVA, the study by Grutters et al. [30] demonstrated that EQ-5D failed to detect significant differences by hearing loss severity groups, whereas HUI3 detected did. Another study found that EQ-5D differentiated the group with the most severe hearing loss but not groups defined by milder levels of deafness [40]. Barton et al. [41] reported that HUI3 mean scores were different between moderate, severe, profound1, profound2 and implanted groups defined by the average of pure-tone air-conduction thresholds at the frequencies 0.5, 1, 2 and 4 kHz in the better hearing ear, although no statistical test was reported. Palmer et al. [42] showed that HUI3 successfully discriminated between people with hearing aids (0.76) and without hearing aids (0.58) at 6 months (*p* < 0.001) and 12 months after intervention (*p* < 0.1) using *t* test. Similarly, HUI3 discriminated 2 groups of patients with cochlear implant and with normal hearing aids where the hearing loss severity of these 2 groups was different according to their pure-tone average. In a study comparing HUI3 and QWB in hearing loss, both scores declined with the degree of hearing loss for children who did not have a cochlear implant with a much greater extent for HUI3 than QWB [28]. A further study of the HUI3 found that it did not differentiate between groups defined according to unilateral or bilateral implantation [27]. However, this finding was also reflected in the VAS measure and may reflect that the additional impact of bilateral implantation in this group is small.

#### Convergence

Three studies presented data for an assessment of convergence of EQ-5D and HUI3. HUI3 showed poor correlation with two speech perception tests; however, a hearing-specific quality of life measure also showed similar results [43]. Gruters et al. [30] reported a moderate correlation between EQ-5D and HUI3. Barton et al. [41] reported a regression analysis and showed that CI (grouped by age at implantation and duration of use), the average of pure-tone air-conduction thresholds at the frequencies 0.5, 1, 2 and 4 kHz in the better hearing ear, and gender were significant predictor of HUI3 (*p* < 0.01) in a large cross-sectional study. HUI3 scores apart from this, no other papers reported correlations between health-related quality of life measures with clinical indicators of hearing loss. Barton et al. [24] reported strong correlations between EQ-5D, HUI3 and SF-SD in their study.

### Responsiveness of GPBMs in hearing impairment

Twelve papers involved a total of 8 studies that provided adequate information to allow an assessment of responsiveness of EQ-5D and/or HUI3 (see Table 4). Only two studies were specifically designed to examine responsiveness of different measures and responsiveness indices such as effect size and standard response mean were reported [38, 39].

**Table 4:** Summary of responsiveness for EQ-5D, HUI3 and SF-6D in hearing impairments

Study	Instruments	Methods	Results				
				Mean change	SD	ES	SRM
Gruters et al. [30]	EQ-5D (UK and Dutch tariff), HUI2 and HUI3	Mean change of scores after hearing aid fitting, ES and SRM	EQ-5D United Kingdom	0.01	0.13	0.05	0.05
			EQ-5D Dutch	0.00,	0.12	0.03	0.02
			HUI2	0.07**	0.13	0.64	0.55
			HUI3	0.12**	0.18	0.57	0.66

			Measure	Before CI	After CI	Mean change
Lee et al. [33]	EQ-5D, QWB, VAS, HUI3	Paired *t*-test for change of scores after CI for EQ-5D, QWB,VAS, HUI and its dimensions.	EQ-5D	0.52	0.78	0.26*
			VAS	0.27 (0.11–0.18)	0.6 (0.45–0.75)	0.33*
			QWB	0.45 (0.3–0.6)	0.61 (0.47–0.75)	0.16*
			HUI	0.29 (0.16–0.42)	0.65 (0.55–0.76)	0.36*
			Vision	0.99 (0.98–1)	0.99 (0.98–1)	0
			Hearing	0.68 (0.63–0.74)	0.87 (0.85–0.9)	0.19*
			Speech	0.95 (0.9–1)	0.99 (0.97–1)	0.04
			Ambulation	0.99 (0.97–1)	0.98 (0.96–1)	−0.1
			Dexterity	1 (1–1)	1 (1–1)	0
			Emotion	0.81 (0.7–0.92)	0.95 (0.91–0.99)	0.14*
			Cognition	0.99 (0.97–1)	0.98 (0.96–1)	−0.01
			Pain	0.96 (0.92–1)	0.95 (0.91–0.99)	−0.01

				ACHA (*n* = 36)		CBHA (*n* = 20)	
				Mean change	ES	Mean change	ES
Hol et al. [38]	EQ-5D, EQ-5D responses, VAS, HHDI and SF-36	Change and ES of EQ-5D, EQ-5D responses, VAS, HHDI domains and SF–36 domains after BAHA (bone-anchored hearing aid).	Mobility	0.02	−0.04	0.15	−0.3
			Self-care	0	0	−0.1	0.28
			Usual activity	−0.03	0.05	−0.05	0.08
			Pain	−0.02	0.04	0.15	−0.28
			Anxiety	0.16	−0.3	−0.06	0.13
			EQ-5D index	−0.01	0.06	−0.01	0.05
			VAS	2.7	0.17	−1.6	0.1
			HHDI				
			Disability	−5.0*	0.79	−10.2*	1.42
			Handicap	−5.4*	0.86	−5.6	0.79*
			SF-36				
			Physical functioning	−0.5	0.02	1.4	−0.06
			Role limitation (physical)	−2.6	0.06	−3.8	0.09
			R Role limitation (emotional)	−3.0	0.07	−13.4	0.33
			Mortality	−0.5	0.02	0.2	−0.01
			Mental health	5.5	−0.28	5.8	−0.36
			Social functioning	5.2	−0.19	1.6	−0.09
			Pain	4.5	−0.18	−5.9	0.24
			General health	−0.4	−0.18	−1.5	0.07

				T0	T1	T2
Joore et al. [31, 32]; Joore [34, 35]	EQ-5D responses, EQ-VAS, ADPI, hearing VAS, SF-36 social domain, AI(Amsterdam Inventory	Change of scores of different measures after hearing aid fitting	ADPI			
			Hearing VAS	0.51	0.77*	0.78*
			Detection of sound	2.38	2.84*	2.87*
			Intelligibility in quiet	1.91	2.87*	2.94*
			Intelligibility in noise	1.95	2.51*	2.35*
			Auditory localization	2.15	2.62*	2.66*
			Distinction of sound	2.38	2.84*	2.87*
			EQ-5D			
			Mobility	2.63	2.68	2.67
			Self-care	2.91	2.94	2.90
			Daily activity	2.81	2.78	2.78
			Pain	2.53	2.55	2.58
			Feeling	2.77	2.91*	2.86
			EQ-5D VAS	0.69	0.71	0.71
			SF-36 social dimension	9.15	9.61	9.69*
			Visit received last month	1.7	1.54	1.64
			Visits paid last month	2.59	2.71	2.64
			AI			
			Discrimination of sounds	3.74	2.2*	1.72*
			Intelligibility in noise	7.67	2.83*	2.67*
			Intelligibility in quiet	7.17	2.64*	2.48*
			Auditory localization	5.1	3*	2.23*
			Distinction of sound	4.55	1.4*	1.14*

				Before fitting	6 months after fitting	95 % CI of difference
Vuorialho et al. [36, 39]	EQ-5D, VAS, HHIE, SRT and WRS	Mean change and statistical test (paired *t*-test or Wilcoxon signed ranks tests) for different measures after hearing aid	SRT	37.9	26.4	
			WRS	92.2	95.6	
			HHIE-S	28.7	12.7	14.2*–*17.8 **
			VAS (SD)	61 (17.9)	65 (16.3)	(−7.1)–(−0.8)**
			EQ-5D index (SD)	0.7 (0.19)	0.7 (0.18)	
			% Reported problems in EQ-5D dimensions			
			Mobility	44.9	54.4	
			Self-care	19.4	15.3	
			Usual activity	45.9	43.9	
			Pain	71.4	62.2	
			Anxiety	17.4	20.4	

				Pre-CI	Post-CI	Change
Cheng et al. [37]	HUI3, VAS, TTO	Perceived change scores; Correlations between change scores	VAS	0.59	0.86	0.27*
			TTO	0.75	0.97	0.22*
			HUI3	0.25	0.64	0.39*
			Hearing	0.65	0.86	0.22*
			Speech	0.80	0.93	0.13*
			Emotion	0.96	0.99	0.03
			Cognition	0.94	0.97	0.03
			Ambulation	0.98	0.99	0.01
			Version	0.98	0.98	0
			Pain	1	1	0
			Dexterity	0.99	0.99	0
			(*n* = 78 (VAS) *n* = 40 (TTO) *n* = 22 (HUI3))			
			Pearson correlations between change scores:			
			VAS/TTO: 0.57 (*n* = 49); VAS/HUI: 0.44 (*n* = 22); TTO/HUI:0.48 (*n* = 15)			

					Group1(*n* = 37)		Group3 (*n* = 22)	
Damen et al. [43]	HUI3, NCIQ	Statistically significant difference between scores of different instruments and their sub-domains pre-/after CI	NCIQ	Pre-CI−	98CI+	04 CI+	98 CI−	04 CI+
			SPB	3.2	65.5*	60.7	10.0	63.5*
			SPA	14.6	55.2*	54.4	14.6	51.7*
			Speech					
			Production	60.5	83.3*	83.3	68.8	80.3**
			Self-esteem	43.0	67.7*	66.8	43.6	69.4*
			Activity	50.0	75.1*	73.6	45.0	71.7*
			Social					
			Interactions	53.7	74.5	63.7*	42.0	60.6*
			HUI 3 utility	0.32	0.64*	0.37	0.38	0.53**
			Vision		93.9	93.7	95.7	93.7
			Hearing		56.6	55.1	13.8	59.2*
			Speech		95.3	94.2	90.1	94.4
			Ambulation		98.7	96.8	96.3	92.6
			Dexterity		98.2	97.9	96.4	98.9
			Emotion		94.9	91.5	90.0	97.6*
			Cognition		96.9	95.8	96.3	84.2
			Pain		93.1	87.8*	91.9	85.1

				Unilateral(*n* = 20)	Bilateral(*n* = (30))	Mann–Whitney test	
				Median IQR	Median IQR	z	*p* value
Lovett et al. [27]	HUI3, VAS, SSQ	Gain in scores of different measures	SSQ speech				
			Section	5.88	7.53	−2.06	0.04
			SSQ				
			Section	4.85	7.47	−3.71	<0.001
			SSQ qualities				
			Section	7.16	7.6	−1.78	0.08
			VAS	0.9	0.91	−1.41	0.16
			HUI 3	0.78	0.83	−0.13	0.91

*EQ*-*5D* euro qol 5 dimension, *HUI2* Health Utility Index 2, *HUI3* Health Utility Index 3, *ES* effect size, *SRM* standard response mean, *CI* cochlear implant, *VAS* Visual Analogue Scale, *HHDI* Hearing Handicap and Disability Index, *SF*-*36* Short-form 36, *BAHA* bone-anchored hearing aid, *ACHA* used air-conduction hearing aid, *CBHA* conventional bone-conduction hearing aid, *NCIQ* the Nijmegen cochlear implant questionnaire, *ADPI* Audiological Disabilities Preference Index, *HHIS*-*S* hearing handicap inventory for the elderly, *SSQ* speech, spatial and qualities of hearing scale for parents, *NVA* test an open speech recognition test, *AN* test assess suprasegmental identification, a closed-set spondee identification test and a closed-set number of syllables test, *DEPTA* better ear pure-tone average hearing loss for the frequencies 1,000, 2,000 and 4,000 Hz, NU-6, Northwestern University 6 word test, *BEHL* better ear hearing levels over the frequencies 0.5–4 kHz, *SRT* speech reception thresholds, *WRS* word reception scores (%), *PTA* ear-specific and bilateral four-frequency pure-tone averages, *APHAB* abbreviated profile of hearing aid benefit

* *p* < 0.05, ** *p* < 0.01

#### Comparison between GPBMs

Comparison of mean change scores of different GPBMs using statistical tests were reported by Gruter et al. [30] and Lee et al. [33] (See below Table 4 for details). Gruter et al.’s study found that HUI2 and HUI3 detected statistically significant change after cochlear implant fitting (0.07 and 0.12, respectively), whereas change scores of EQ-5D were smaller and not statistically significant (0.01). In terms of effect size, HUI2 and HUI3 were high (0.57 and 0.64, respectively), whereas the change in EQ-5D was very small (0.02 and 0.05 for both UK and Dutch tariffs). The study by Lee et al. demonstrated that the increase in scores of the GPBMs, including EQ-5D (0.26), VAS (0.33), HUI3 (0.36) and QWB (0.16), was all statistically significant following cochlear implantation. For HUI3 dimensions, score increases for hearing (0.19) and emotion (0.14) was statistically significant (*p* < 0.05), whereas non-significant for other dimensions. The results suggest that the EQ-5D was responsive in capturing larger improvements in hearing as in the study by Lee et al. but was not able to capture the smaller levels of improvement shown in the study by Grunter et al.

#### Responsiveness of EQ-5D

Eight papers reported the responsiveness of EQ-5D without the other 2 generic measures by comparing them with EQ-VAS, hearing VAS or other hearing-specific measures, which involved a total of 4 separate studies. In these studies, no statistically significant changes before and after the hearing intervention were detected by the EQ-5D [31, 32, 34, 35, 38, 39] and the effect size where reported was very low [38]. Whereas statistically significant improvements were shown in VAS scores [32, 34–36, 39], and condition-specific measures such as two sub-domains (disability and handicap) of Hearing Handicap and Disability Index [38], overall scores of the Hearing Handicap Inventory for the Elderly and its 2 sub-domain scores [36, 39], and the 5 questions of Amsterdam Inventory and Audiological Disabilities Preference Index [35]. Joore’s study reported the self-perceived SF-36 social functioning which was significantly improved after hearing aid fitting in long term.

#### Responsiveness of HUI3

Three papers reported responsiveness of HUI3 without the other 2 generic measures, comparing with VAS/TTO and hearing-specific measures [27, 37, 43]. Cheng et al. found that the change of HUI3 overall score (0.39) was higher than both VAS score (0.27) and TTO (0.22) after cochlear implant fitting, but all were statistically significant (*p* < 0.1). Only the change scores of hearing and speech dimension of HUI3 were significant and the hearing dimension had the biggest change score while scores of other dimensions were stable over time. Moderate correlations (around 0.48) between change scores of VAS, TTO and HUI3 were found [43].

## Discussion and conclusions

The 18 papers (14 studies) included in this review provide useful information to assess the validity and responsiveness of GPBMs for use in hearing impairment. A summary of the overall performance of the 3 GPBMs is provided in Table 5. There was heterogeneity in the studies reviewed, in terms of study design, patient populations, which needs to be taken into account when interpreting the findings.

**Table 5:** Overall performances of EQ-5D, HUI3 and SF-6D in hearing impairment

	Known group (Severity)		Known group (case–control)		Known group (other)		Correlation	Responsive (change)	
	Cons	Sig	Cons	Sig	Cons	Sig		Cons	Sig
*EQ*-*5D*									
Grutters et al. [30]					√	√	Moderate	×	×
Sach and Barton [40]	Severe √ Mild ×	√×			√	√			
Lee et al. [33]								√	√
Hol et al. [38]								×	×
Joore et al. [31, 32]; Joore [34, 35]								?	×
Vuorialho et al. [36, 39]								×	×
*HUI 3*									
Barton et al. [24]	√	N/R							
Bichey et al. [29]	√	N/R							
Grutters et al. [30]	√	√					Moderate	√	√
Palmer et al. [42]	√	√							
Smith-Olinde et al. [28]	√	N/R							
Lee et al. [33]								√	√
Cheng et al. [37]								√	√
Damen et al. [43]							Moderate (sig)	√	√
Lovett et al. [27]	√	×						√	×
*SF*-*6D*									
Barton et al. [24]							Moderate to strong		

*Cons* consistent with other measures, *sig* statistically significant

√: Yes ?: Mixed evidence ×: No N/R: no report

Overall, the HUI3 was the most commonly used measure in the studies. In all 6 cases, the HUI3 detected difference between groups defined by their severity of hearing impairment and 4 out of 5 cases detected statistically significant changes as a result of intervention. Differences picked up by the HUI3 were driven by the hearing dimensions, and also, in some cases, the speech dimension and the emotion dimension. On the other hand, the findings of the review suggested relatively poor responsiveness of EQ-5D in this condition as in 4 out of 5 cases EQ-5D failed to detected change. The only study that allowed an assessment of known groups using the EQ-5D suggested it only had weak ability to discriminate difference between severity groups. Only one study involved the SF-6D, thus the information is too limited to conclude on its performance. Converting published mean SF-36 scores into SF-6D would not help since psychometric testing requires individual level data.

Two studies reported validity of EQ-5D where the results were mixed when the groups were defined by severity of hearing impairment [40]. In terms of responsiveness, EQ-5D did not demonstrate statistically significant changes after hearing aid fitting but there were statistically significant changes detected by clinical indicators or condition-specific measures. The EQ-5D appears to reflect less, or often no, change/difference compared to the HUI3 and clinical measures. Possible reasons for this include the EQ-5D not capturing important effects of hearing on quality of life, or that the changes/differences in hearing have little impact on overall quality of life (e.g. because the level of change is small or people have adapted to their hearing loss and value other aspects of health more). Also, the use of clinical measures or else for grouping hearing impairment severity may be regarded as poor indicators to use for testing construct validity. However, given that significant differences were found for HUI3 and hearing-specific health-related quality of life measures, it suggests that the lack of significant differences for EQ-5D is a concern.

There were 2 exceptions to the poor performance of EQ-5D: one study demonstrated a statistically significant improvement in EQ-5D index scores after cochlear implantation and in another study, the EQ-5D differentiated between severe hearing loss but not in different levels of milder hearing loss. Although EQ-5D utility indices remained stable over time, 1 study showed that the proportion of respondents who reported problems for dimensions of EQ-5D increased or decreased. Another study treated responses of EQ-5D dimension as continuous variables to compare mean responses before and after intervention, which was judged to be problematic for analysis. There were 2 studies where the HUI3 reflected differences, but much smaller differences were found in the EQ-5D utilities.

It is perhaps unsurprising that HUI3 performs well as it explicitly includes a hearing dimension. The lack of a direct reference to an impairment or symptom in EQ-5D does not mean that, by definition, its effects will not be captured; however, this review suggests that EQ-5D may perform poorly in this particular type of condition. A five-level version of the EQ-5D has recently been developed and this may overcome the problem if it is simply one of the sensitivity. However, it may be related to a lack of relevant dimensions to pick up the impact of hearing loss. Evidence is required using the 5-level version to confirm this hypothesis. Another approach to the problem might be to use a condition-specific preference-based measure for hearing like the one developed by Yang et al. for asthma [44] or Rowen et al. in cancer [45]. The problem with using condition-specific measures is that they may miss important side effects of treatment and the values may exaggerate the impact of the conditions due to focusing effects by member of the general public [46]. This is the reason for ongoing research into developing bolt-ons to the EQ-5D to cover those dimensions that appear to be missing for cognition in EQ-5D [47], and in the same way, one could be developed for hearing. In the meantime, the best option appears to be to use HUI3.

It cannot always be assumed that a generic measure should reflect the change of health states which a condition-specific measure detects. Disease- or condition-specific measures are tailored to the condition of interest and are therefore more focussed on the condition of interest and may be more sensitive to change. However, they may not capture the broader impacts of the condition on health-related quality of life. In addition, the general population (or indeed the patients) may not regard the change as sufficiently important when valuing health. Interestingly, in this study, not only GPBMs, but also the hearing-specific measures showed poor correlations with clinical indicators. This emphasises the importance of including patient-reported outcome measures in the evaluations of health care interventions. In addition, preference-based measures can reflect how changes in health states are valued, in relation to other aspects of health.

This is the first-time information on the validity and responsiveness of GPBMs that have been comprehensively reported and analysed in hearing impairment. This paper reports important findings for the use of GPBMs of health to compare the impact of hearing loss on health-related utility. The results indicate that HUI3 is an appropriate measure for use in hearing impairment given its good performance of validity and responsiveness. EQ-5D was not responsive to modest changes in hearing impairment, and the limited evidence suggested it has weak validity in this condition. Very little evidence was found for SF-6D.

## Appendix 1: Search strategy used for the hearing review in Medline

1. (euroqol or euro qol or eq5d or eq 5d or eq-5d or (euro adj qol) Or eur adj qual) or (eq adj 5d)).mp
2. (hui3 or hui 3 or health utilities index mark 3 or health utilities mark three or hui III or huiIII).mp
3. (sf6D or sf 6D or short form 6D or shortform 6D or sf six D or sfsixD or shortform six D or short form sixD or sf-6d or 6d or 6-d or 6 dimension).mp
4. (hearing disorder or dysacusis or paracousis or paracusis or Distorted hearing).mp
5. (hearing loss or hearing complaints or hearing aids or cochlear implants).mp. [mp = title, original title, abstract, name of substance word, subject heading word, unique identifier]
6. hearing disorders/
7. 1 or 2 or 3
8. 4 or 5 or 6
9. 7 and 8

## Abbreviations

ADPI: Audiological Disability Preference Index
AI: Amsterdam Inventory
BEPTA: Better ear unaided pure-tone average
CI: Cochlear implant
EQ-5D: Euroqol 5 dimensions
EQ-VAS: Euroqol Visual Analogue Scale
ES: Effect size
GPBMs: Generic preference-based measures of health
HHDI: Hearing Handicap and Disability Index
HHIE-S: Hearing Handicap Inventory for the Elderly
HRQoL: Health-related quality of life
HSUVs: Health state utility values
HUI3: Health Utilities Index 3
ICC: Intra-class correlation coefficients
LVAS: Large vestibular aqueduct syndrome
NCIQ: Nijmegen cochlear implant questionnaire
NICE: National institute for health and clinical excellence
QALY: Quality-adjusted life year
QWB: Quality of well-being
SF-6D: Short-form 6 dimensions
SF-36: Short-form 36
SF-12: Short-form 12
SG: Standard gamble
SRM: Standard response mean
SSQ: Speech spatial and qualities of hearing scale for parents
TTO: Time trade-off
VAS: Visual Analogue Scale
